# Predicting the resting metabolic rate of young and middle-aged healthy Korean adults: A preliminary study

**DOI:** 10.20463/pan.2020.0002

**Published:** 2020-03-31

**Authors:** Hun-Young Park, Won-Sang Jung, Hyejung Hwang, Sung-Woo Kim, Jisu Kim, Kiwon Lim

**Affiliations:** 1 Department of Sports Medicine and Science of Graduated School, Konkuk University, Seoul Republic of Korea; 2 Physical Activity and Performance Institute (PAPI), Konkuk University, Seoul Republic of Korea; 3 Department of Physical Education, Konkuk University, Seoul Republic of Korea

**Keywords:** RMR, fat-free mass, age, regression coefficient, algorithm model, estimation equation

## Abstract

**[Purpose]:**

This preliminary study aimed to develop a regression model to estimate the resting metabolic rate (RMR) of young and middle-aged Koreans using various easy-to-measure dependent variables.

**[Methods]:**

The RMR and the dependent variables for its estimation (e.g. age, height, body mass index, fat-free mass; FFM, fat mass, % body fat, systolic blood pressure, diastolic blood pressure, mean arterial pressure, pulse pressure, and resting heart rate) were measured in 53 young (male n = 18, female n = 16) and middle-aged (male n = 5, female n = 14) healthy adults. Statistical analysis was performed to develop an RMR estimation regression model using the stepwise regression method.

**[Results]:**

We confirmed that FFM and age were important variables in both the regression models based on the regression coefficients. Mean explanatory power of RMR_1_ regression models estimated only by FFM was 66.7% (R^2^) and 66.0% (adjusted R^2^), while mean standard errors of estimates (SEE) was 219.85 kcal/day. Additionally, mean explanatory power of RMR_2_ regression models developed by FFM and age were 70.0% (R^2^) and 68.8% (adjusted R^2^), while the mean SEE was 210.64 kcal/day. There was no significant difference between the measured RMR by the canopy method using a metabolic gas analyzer and the predicted RMR by RMR_1_ and RMR_2_ equations.

**[Conclusion]:**

This preliminary study developed a regression model to estimate the RMR of young and middle-age healthy Koreans. The regression model was as follows: RMR_1_ = 24.383 × FFM + 634.310, RMR_2_ = 23.691 × FFM - 5.745 × age + 852.341.

## INTRODUCTION

Resting metabolic rate (RMR) is the total number of calories burned when the body is completely at rest. It is proportional to the lean body mass and decreases by approximately 0.01 kcal/min for each 1% increase in the body fat^1^. The total energy expenditure in 24 hours consists of RMR, physical activity energy expenditure (PEE), and diet-induced thermogenesis (DIT). The RMR represents approximately 60-75% of daily energy expenditure (DEE) in a 70 kg person^2,3^, accounting for the largest contribution to the 24-hour energy expenditure. It consequently has a large impact on the regulation of body composition and energy balance. An abnormally high RMR is associated with pathologic and inflammatory conditions. It also tends to decrease with aging, with a low RMR playing an important role in the pathogenesis of obesity and age related chronic diseases^4-6^. Therefore, an accurate measurement of the RMR is very important.

Methods for measuring the RMR include direct and indirect calorimetry, with the latter being used commonly due to a more efficient measurement. It is further divided into two methods, one using doubly labeled water; and the other using a human metabolic chamber, a hood-, or a mask system^7-9^. However, both the methods require tedious procedures and are time- and cost- consuming. Thus, many researchers have developed various regression models for RMR estimation^2,9-14^. Determining the contribution of the RMR to the DEE is an important calculation for understanding, developing, and executing body weight- related interventions^3,4^. For example, RMR estimation regression model is applied to determine the target energy intake in weight loss programs, develop dynamic prediction models of weight gain and loss, identify the patients with potential metabolic abnormalities, design public health programs promoting obesity prevention in diverse populations, and assess the potential energy deficits in metabolically stressed patients^4^.

The previous regression models were developed using only height, age, weight, and fat-free mass (FFM) and hence did not have a high correlation and regression rate. The Harris–Benedict regression model^10^ was the most commonly used, but only 50-75% of the RMR variability could be explained by this equation. Its major disadvantage was overestimating RMR by at least 5%. Additionally, RMR was estimated only by age, height, and weight and the regression rate, R^2^ value, did not exceed 0.7. Previous attempts to overcome these shortcomings failed to show high regression rates^11-14^, as they also estimated RMR with only age, height, weight, and lean body mass. Therefore, developing a regression model, with a higher regression rate using various dependent variables, is important for accurate RMR measurements.

The present study was a preliminary study and it aimed to evaluate the Korean adults (males and females) to generate regression equations to predict the RMR from age, height, body mass index (BMI), FFM, fat mass, % body fat, systolic blood pressure (SBP), diastolic blood pressure (DBP), mean arterial pressure (MAP), pulse pressure (PP) and resting heart rate (HR).

## METHODS

### Subjects

Fifty three young (males = 18, females = 16) and middle-aged (males = 5, females = 14) healthy adults were included in the present study <Table 1>^7^. All subjects were of Korean origin, with a stable weight for at least 3 months prior to the measurements, and without a history of thyroid disease, diabetes mellitus-I or II, cardiovascular disease, or severe hypertension in the past 6 months. There was also no history of orthopedic disease or other medical issues over the past year in the pre-screening surveys. All the subjects received a medical clearance for their participation and were explained about the purpose, procedures, and potential risks of the study. All proceedings of the study were approved by the Institutional Review Board of Konkuk University (7001355-201903-HR-305) in Korea and were conducted according to the Declaration of Helsinki. All subjects arrived at the laboratory early in the morning (8:00 AM) after overnight fasting (≥ 8 hour) and rested for 30 minutes, after which their blood pressure, body composition, and resting HR was measured, followed by the RMR measurement. All the subjects were instructed to sleep for at least 8 hours before the RMR measurement and to stay awake during the process. If they fell asleep, their shoes or toes were squeezed to keep them awake.

**Table 1.** Characteristics of subjects.

	Both (n=53) (Range)	Males (n=23) (Range)	Females (n=30) (Range)
Age (yrs)	32.15±12.08 (19-58)	29.22±10.10 (19-55)	34.40±13.12 (19-58)
Body height (cm)	167.51±9.95 (151.0-189.9)	176.60±7.16 (164.4-189.9)	160.55±4.79 (151.0-170.5)
Body weight (kg)	63.75±12.71 (47.2-90.9)	75.51±9.25 (51.8-90.9)	54.74±5.55 (47.2-69.1)
Body mass index (kg/m^2^)	22.50±2.42 (18.8-28.1)	24.15±2.04 (19.2-27.8)	21.24±1.87 (18.8-28.1)
Fat-free mass (kg)	48.11±12.63 (32.2-74.0)	60.97±7.40 (43.1-74.0)	38.25±3.55 (32.2-50.4)
Fat mass (kg)	15.53±4.53 (5.4-28.4)	14.39±4.75 (5.4-25.3)	16.41±4.22 (7.5-28.4)
Percent body fat (%)	25.01±7.62 (7.1-41.1)	18.89±5.08 (7.1-30.6)	29.71±5.65 (14.6-41.1)
Systolic blood pressure (mmHg)	117.41±12.69 (89.0-142.5)	124.42±9.27 (103.5-142.5)	112.02±12.42 (89.0-142.0)
Diastolic blood pressure (mmHg)	70.43±10.52 (51.5-92.5)	73.94±9.66 (54.0-92.5)	67.75±10.51 (51.5-88.0)
Mean arterial pressure (mmHg)	86.09±10.62 (64.17-108.0)	90.77±9.00 (70.5-108.0)	82.51±10.50 (64.17-106.0)
Pulse pressure (mmHg)	46.97±8.10 (27.0-66.5)	50.50±6.64 (39.5-62.5)	44.27±8.17 (27.0-66.5)
Heart rate (beat/min)	68.99±9.73 (48.5-94.5)	67.17±11.01 (50.0-92.5)	70.38±8.55 (48.5-94.5)
RMR (kcal/day)	1807.43±377.11 (1261.40-2735.35)	2165.21±265.26 (1632.37-2735.35)	1533.14±149.24 (1261.40-1823.85)

Note. Values are expressed as mean ± SD. RMR = resting metabolic rate.

### Height and body composition

Height, BMI, body weight, FFM, fat mass, and % body fat were measured using the bioelectrical impedance analysis equipment (Inbody 770, Inbody, Seoul, Korea). All subjects fasted overnight prior to body composition measurement. The subjects wore lightweight clothing and were asked to remove any metal items.

### Blood pressure and resting heart rate

After all the subjects were sufficiently rested for more than 20 min, their blood pressure was measured twice using an autonomic blood pressure monitor (HBP-9020, Omron, Tokyo, Japan) and the average value was used for analysis. The blood pressure parameters measured were SBP, DBP, MAP, and PP. The resting HR was measured using an autonomic HR monitor (V800, Polar, Helsinki, Finland).

### Resting metabolic rate

All subjects fasted overnight, prior to the measurement of the RMR by indirect calorimetry using a metabolic gas analyzer (Quark CPET, Cosmed, Rome, Italy) with a flow-dilution canopy hood system. Calibration was performed using the calibration gas (16% O_2_ and 5% CO_2_) before the measurement. All the RMR testing procedures were performed in a 9 m (width) × 7 m (length) × 3 m (height) chamber with a temperature of 23 ± 1 °C and a humidity of 50 ± 5%, regulated by the environmental control chamber (NCTC-1, Nara Controls, Seoul, Korea). Subjects were asked to limit their physical activity and abstain from alcohol intake one day before the measurement. The subjects took a rest for 30 min, prior to the measurement. The RMR was measured in a supine position for 30 min, and the average value of the last 25 min was used for the analysis.

### Statistical Analysis

The means and standard deviations were calculated for all the measured parameters. The Shapiro-Wilk test verified the normal distribution of all the outcome variables. To perform the linear regression analysis, we verified the independent variables by checking the regression coefficient (*β*-value). Regression analysis using the stepwise method was used to predict the RMR from age, height, BMI, FFM, fat mass, % body fat, SBP, DBP, MAP, PP, and HR. A two-tailed student’s paired t-test was used to detect differences between measured and predicted RMR. Bias was calculated as the difference between measured and predicted RMR. The authors rigorously conformed to the basic assumptions of a regression model (linearity, independency, continuity, normality, homoscedasticity, autocorrelation, and outlier). Statistical Package for the Social Sciences (SPSS) version 24.0 (IBM Corporation, Armonk, NY, USA) was used for the statistical analysis and the level of significance (*p value*) was set at 0.05.

## RESULTS

### Check outlier data

We verified the absolute value of the standardized residual as ≥ 3 to delete the outlier data. There was no outlier data in the RMR prediction model. The correlation between the measured RMR and dependent variables is shown in Table 2. We estimated the regression model using the stepwise method.

**Table 2.** Correlation between dependent variables and measured RMR for estimating regression model.

		RMR (kcal/day)		
		Both (n=53)	Males (n=23)	Females (n=30)
Age (yrs)	Correlation *p*-value	-.284* .039	-.214 .327	-.212 .262
Body height (cm)	Correlation *p*-value	735* .000	.171 .434	.200 .289
Body weight (kg)	Correlation *p*-value	.743* .000	.293 .176	-.065 .732
Body mass index (kg/m^2^)	Correlation *p*-value	.536* .000	.275 .204	-.231 .218
Fat-free mass (kg)	Correlation *p*-value	.817* .000	.292 .176	.168 .375
Fat mass (kg)	Correlation *p*-value	-.191 .171	.142 .517	-.233 .215
Percent body fat (%)	Correlation *p*-value	-.631* .000	.036 .870	-.259 .167
Systolic blood pressure (mmHg)	Correlation *p*-value	.408* .002	.236 .278	-.250 .183
Diastolic blood pressure (mmHg)	Correlation *p*-value	.279* .043	.295 .172	-.220 .242
Mean arterial pressure (mmHg)	Correlation *p*-value	.346* .011	.292 .176	-.246 .191
Pulse pressure (mmHg)	Correlation *p*-value	.277* .045	-.099 .653	-.097 .610
Heart rate (beat/min)	Correlation *p*-value	-.149 .288	.004 .986	-.060 .751

Note. *Significant correlation between measured RMR and dependent variables, *p* < 0.05. RMR = resting metabolic rate.

### Significance of regression models and the independent variables

We verified the significance of each model using the F-test; and used the *t*-test to verify the significance of the regression coefficients of the independent variables.

Individual regression models for males and females could not be developed. This was due to the small sample size and the absence of a significant correlation between the dependent variables and the RMR which resulted in multicollinearity among all the dependent variables.

The regression coefficient of the selected independent variables (age and FFM) was statistically significant <Table 3> when an integrated regression model using the stepwise method for both males and females was developed.

**Table 3.** Significance level of the regression coefficient of the independent variable (age and FFM) for each estimated regression model.

Regression model				
Model	F-value	*p*-value	*t*-value	*p*-value
RMR_1_ = 24.383 × FFM + 634.310	102.005	.000*	10.100 (FFM)	.000* (FFM)
RMR_2_ = 23.691 × FFM - 5.745 × age + 852.341	58.332	.000*	10.160 (FFM)	.000* (FFM)
			-2.357 (age)	.022* (age)

Note. *Statistically significant, *p* < 0.05. RMR = resting metabolic rate, FFM = fat-free mass.

### Performance evaluation of regression models and regression equations

The coefficients of determination (R^2^), adjusted coefficients of determination (adjusted R^2^), and standard errors of estimates (SEE) were calculated for the regression model. The mean explanatory power of RMR_1_ regression models estimated only by FFM was 66.7% (R^2^) and 66.0% (adjusted R^2^), while the mean SEE was 219.85 kcal/day <Table 4>. The mean explanatory power of RMR_2_ regression models developed by FFM and age were 70.0% (R^2^) and 68.8% (adjusted R^2^), while the mean SEE was 210.64 kcal/day <Table 4>.

**Table 4.** Estimated regression equations predicting RMR of young and middle-aged Koreans.

Regression model					
Model	R	R^2^	Adjusted R^2^	*p*-value	SEE
RMR_1_ = 24.383 × FFM + 634.310	.817	.667	.660	.000*	219.85
RMR_2_ = 23.691 × FFM - 5.745 × age + 852.341	.837	.700	.688	.000*	210.64

Note. *Statistically significant, p < 0.05. RMR = resting metabolic rate, SEE = standard error of estimate.

### Difference between measured and predicted RMR of young and middle-aged Korean adults

**Figure 1.**
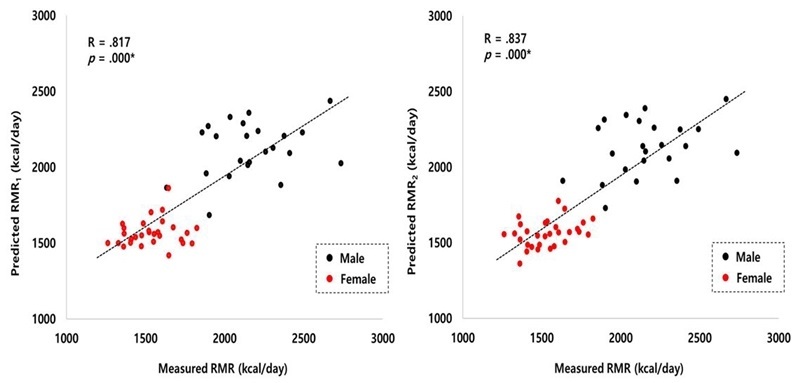
Scatter plot between measured and predicted RMR of young and middle-aged Koreans. Note. RMR = resting metabolic rate.

In the present study, there was no significant difference between the measured RMR by canopy method, using a metabolic gas analyzer, and the predicted RMR by RMR_1_ and RMR_2_ equations. The mean bias between the measured RMR and the predicted RMR_1_ and RMR_2_ equations was + 0.02 kcal/day and -0.01 kcal/day, respectively <Table 5>. The measured and the predicted RMR showed a similar average value, and their correlation coefficients also showed a significant correlation (measured RMR and predicted RMR_1_: R = 0.817, *p* = 0.000, measured RMR and predicted RMR_2_: R = 0.837, *p* = 0.000) <Figure 1>.

**Table 5.** Measured and predicted RMR of young and middle-aged Koreans.

Model	Variables	Mean	S.D.	Bias	*t*-value	*p*-value
RMR_1_	Predicted RMR (kcal/day)	1807.41	307.91	0.02	.001	.999
	Measured RMR (kcal/day)	1807.43	377.11			
RMR_2_	Predicted RMR (kcal/day)	1807.44	315.52	-0.01	.000	1.000
	Measured RMR (kcal/day)	1807.43	377.11			

Note. RMR = resting metabolic rate. Bias = measured RMR – predicted RMR.

## DISCUSSION

A preliminary study was conducted to develop a regression model for estimating the RMR of healthy young and middle-aged Korean adults using various easy-to-measure dependent variables. Based on the data obtained, our study developed two regression models (RMR_1_ = 24.383 × FFM + 634.310, RMR_2_ = 23.691 × FFM - 5.745 × age + 852.341).

Before developing a regression model to estimate the RMR, it is important to eliminate the outliers as they increase the forecast errors. In a regression analysis, the determination of outliers uses the absolute value of the standardized residual^15^. No outliers were observed in this study. This finding demonstrated a clear linearity between the independent and the dependent variables.

Table 2 shows the correlation between the RMR and the various dependent variables in males, females, and the total sample. Most of the measured variables presented a significant correlation with the RMR (e.g. age, height, weight, BMI, FFM, % body fat, SBP, DBP, MAP, and PP). However, autocorrelation and multicollinearity were observed in all the dependent variables except FFM and age, therefore, two RMR regression models using FFM and age were developed using the stepwise method. The developed equations indicated that 66.7% (RMR_1_ equation) and 70.0% (RMR_2_ equation) of the variance in the criterion variable of RMR was attributable to the variance of the combined predictor or independent variables.

Previously, Harris and Benedict^10^ developed separate RMR estimation models (male = 66.5 + 13.75 × weight + 5.003 × height – 6.775 × age, R^2^ = 0.64, female = 655.1 + 9.563 × weight + 1.850 × height – 4.676 × age, R^2^ = 0.36) for males (n = 136) and females (n = 103). Only 50-75% of the RMR variability could be explained by this equation, with the disadvantage of overestimating the RMR by at least 5%. Further, the RMR was estimated only by age, height, and weight and the regression rate, R^2^ value, did not exceed 0.7. Schofield^12^ developed an RMR estimation model for Italian males based on multiple sample sizes (n = 2879, RMR_1_ = 63.0 × weight + 2896, RMR_2_ = 63.0 × weight - 0.42 × height + 2953). However, the samples had a disproportionate number of Italian military cadets, soldiers, workers, and miners, who did not represent the typical Italian population. These constituted 56% of the 18 - 30 years old male cohort^2^. Additionally, the regression rate of the RMR estimation model was low (RMR_1_: R^2^ = 0.423, RMR_2_: R^2^ = 0.423). Hayter & Henry^16^ developed an RMR estimation model using Schofieldʼs database, which predicted the RMR in Northern Europeans and Americans, except Italians. However, this model also had a regression rate similar to that of Schofield (n = 478, RMR = 51.0 × weight + 3500, R^2^ = 0.449)

Conversely, Roza and Shizgal^11^ re-evaluated the Harris-Benedict equation using the age, height, and weight used in the latter’s estimation model using normal subject data (n = 239) from the study and additional data, which were obtained from the subjects spanning a wider age range (n = 98). As a result, a model with a relatively higher regression rate (male: 88.362 + 13.397 × weight + 4.799 × height - 5.677 × age, R^2^ = 0.77, female: 447.593 + 9.247 × weight + 3.098 × height - 4.330 × age, R^2^ = 0.69) was developed. Also, Mifflin et al.^13^ developed a predictive equation for RMR from the data of 498 healthy subjects, including females (n = 247) and males (n = 251), aged 19-78 yrs (45 ± 14 yrs). Normal weight (n = 264) and obese (n = 234) individuals were studied and the RMR was measured by indirect calorimetry. This model showed high regression rates for both males and females (male: 10 × weight + 6.25 × height – 5 × age + 5 kcal/day, R^2^ = 0.71, female: 10 × weight + 6.25 × height – 5 × age - 161 kcal/day).

The present preliminary study developed an RMR estimation model with a regression rate that was similar to Roza & Shizga^11^ and Mifflin et al.^13^ using FFM and age, with a smaller sample size. This was due to the strict adherence to the basic assumptions of a linear regression model. Compared to a study by Piers et al.^9^, which developed an RMR estimation model using similar sample sizes and independent variables, the present model showed a higher regression rate.

In conclusion, we developed a regression model using FFM and age to estimate the RMR of young and middle-aged healthy Korean adults through preliminary experiments. The developed model was as follows: RMR_1_ = 24.383 × FFM + 634.310, RMR_2_ = 23.691 × FFM - 5.745 × age + 852.341. The bias (RMR_1_ = 0.02, RMR_2_ = -0.01) and correlation (RMR_1_: R = 0.817, RMR_2_: R = 0.837) between the estimated RMR and the measured RMR were reasonable.

However, the present study was a preliminary study and had its limitations. The regression model for individual genders could not be developed due to the small sample size, and a validity test could not be performed. Further research to overcome these limitations is thus recommended.

